# Systematic profiling of polyclonal HIV antibodies and prediction of effector functions

**DOI:** 10.1186/1742-4690-9-S2-P86

**Published:** 2012-09-13

**Authors:** E Brown

**Affiliations:** 1 Dartmouth College, Hanover, NH, USA

## Background

Antibody-dependent effector functions (ADEF) with the ability to recruit the innate immune response may play an important role for the spread of HIV infections. ADEF are mediated by the antibody Fc and depend on antibody class and glycosylation status. We describe the systematic profiling of the Fc regions of HIV-specific antibodies isolated from different patient sera.

## Methods

A LuminexTM-based suspension array was used to capture HIV antibody fractions binding to various HIV antigens on beads and probe them for binding to (1) antibody class-specific binding reagents, (2) Fc receptors, (3) complement proteins and (4) different lectins. The obtained binding profiles are correlated with other measures of effector function and may help to understand which ADEF are crucial to provide protection.

## Results

Subclassing data has been used to correlate patients with enhanced measures of effector function such as phagocytosis or ADCVI. In addition, results of the Fc-gamma-receptor binding assay have been correlated to traditional measures of affinity such as SPR, showing that our method is giving useful results. We have also used epitope-specific reagents like the resurfaced stabilized core form of gp120 to isolate and probe eppitope-specific antibodies.

## Conclusion

We hope to use our system as a means to quickly evaluate antibodies induced in an HIV vaccine setting. Being able to get a rapid profile of the polyclonal antibody response should be a useful predictor of vaccine efficacy. In addition, the assay we have devised is easily customizable, as antigens and receptors can be tailored to fit our interests.

